# A fatal case of disseminated nocardiosis due to *Nocardia otitidiscaviarum* resistant to trimethoprim–sulfamethoxazole: case report and literature review

**DOI:** 10.1186/s12941-022-00511-9

**Published:** 2022-05-16

**Authors:** Mazin Barry, Shahad AlShehri, Ahlam alguhani, Mohammad Barry, Ali Alhijji, Khalifa Binkhamis, Fahad Al-Majid, Fatimah S. Al-Shahrani, Taim Muayqil

**Affiliations:** 1 Division of Infectious Diseases, Department of Internal Medicine, College of Medicine, King Saud University, PO Box 2925, Riyadh, 11461 Saudi Arabia; 2 Infectious Diseases Unit, Internal Medicine Department, Prince Sultan Military Medical City, Riyadh, Saudi Arabia; 3 Infectious Diseases Unit, Internal Medicine Department, King Abdullah Medical City National Guard, Riyadh, Saudi Arabia; 4 Medical Imaging Department, King Abdullah bin Abdulaziz University Hospital, Riyadh, Saudi Arabia; 5 Department of Pathology, College of Medicine, King Saud University, Riyadh, Saudi Arabia; 6 King Saud University Medical City, King Saud University, Riyadh, Saudi Arabia; 7 Division of Infectious Diseases, Faculty of Medicine, University of Ottawa, Ottawa, Canada; 8 Division of neurology, Department of Internal Medicine, College of Medicine, King Saud University, Riyadh, Saudi Arabia

**Keywords:** Nocardiosis, *Nocardia otitidiscaviarum*, Trimethoprim–sulfamethoxazole (TMP–SMX)

## Abstract

**Background:**

Disseminated nocardiosis still causes significant morbidity and mortality and is often caused by *Nocardia asteroides*, *N. basiliensis*, and *N. farcinica* and are often treated with trimethoprim–sulfamethoxazole (TMP–SMX). *Nocardia otitidiscaviarum* (*N. otitidiscaviarum*) rarely causes disseminated disease and resistance to TMP–SMX is even more rare.

**Case presentation:**

A 37-year-old woman with metastatic breast cancer and right ear deafness with recent occupational gardening and manipulating soil, presented to the hospital with first time seizure and multiple skin nodules. Magnetic resonance imaging (MRI) showed ring enhancing lesions, biopsy of the skin and brain lesions grew *N. otitidiscaviarum.* She was empirically treated with TMP–SMX and Imipenem–Cilastatin, however, almost three weeks into therapy, susceptibility results revealed it to be resistant to both antimicrobials, she was subsequently changed to Amikacin, Linezolid, Moxifloxacin, and Doxycycline but ultimately died.

**Conclusions:**

This case report highlights the importance of suspecting a rare *Nocardia* species in patients at risk with proper occupational exposure, moreover, TMP–SMX resistance should be suspected with lack of clinical response, this may have important implications on clinical practice when facing similar infections.

## Background

Disseminated nocardiosis is a rare disease that can be fatal and is often attributed to the three more commonly known species: *Nocardia asteroides*, *N. basiliensis*, and *N. farcinica *[1]. *N. otitidiscaviarum* was first isolated from the mid-ear of a guinea pig and hence its name, it was then isolated from humans as well [2, 3]. Trimethoprim–sulfamethoxazole (TMP–SMX) or Linezolid are the backbone of therapy regimens for nocardiosis. However, a combination therapy is usually required. Resistance to TMP–SMX is rarely reported. We describe a case of an extensively disseminated infection with *N. otitidiscaviarum*, a species that was rarely described as a significant pathogen in the medical literature.

## Case

A 37-year-old woman presented with a generalized tonic–clonic seizure that spontaneously aborted after three minutes with post-ictal incontinence. Over the preceding one month she had noticed multiple nodules under her skin on the right leg, and over her abdomen. They were mildly painful, eventually the overlying skin developed dark discoloration. There was no drainage, and she did not have similar skin lesions in the past. She is a known case of stage 4 invasive ductal carcinoma of the breast with metastasis to the liver, bone, and lungs that was diagnosed two years prior to the current presentation. She was recently on carboplatin/paclitaxel for a total of 8 weeks with the last dose given 3 weeks prior to this presentation. She has not been on corticosteroids in the preceding three months. Furthermore, over the past months prior to her current illness, she had been caring for domestic canaries and gardening frequently without the use of personal protective equipment nor gloves, reporting handling dirt and soil with her bare hands with frequent touching of her face.

**Fig. 1:**
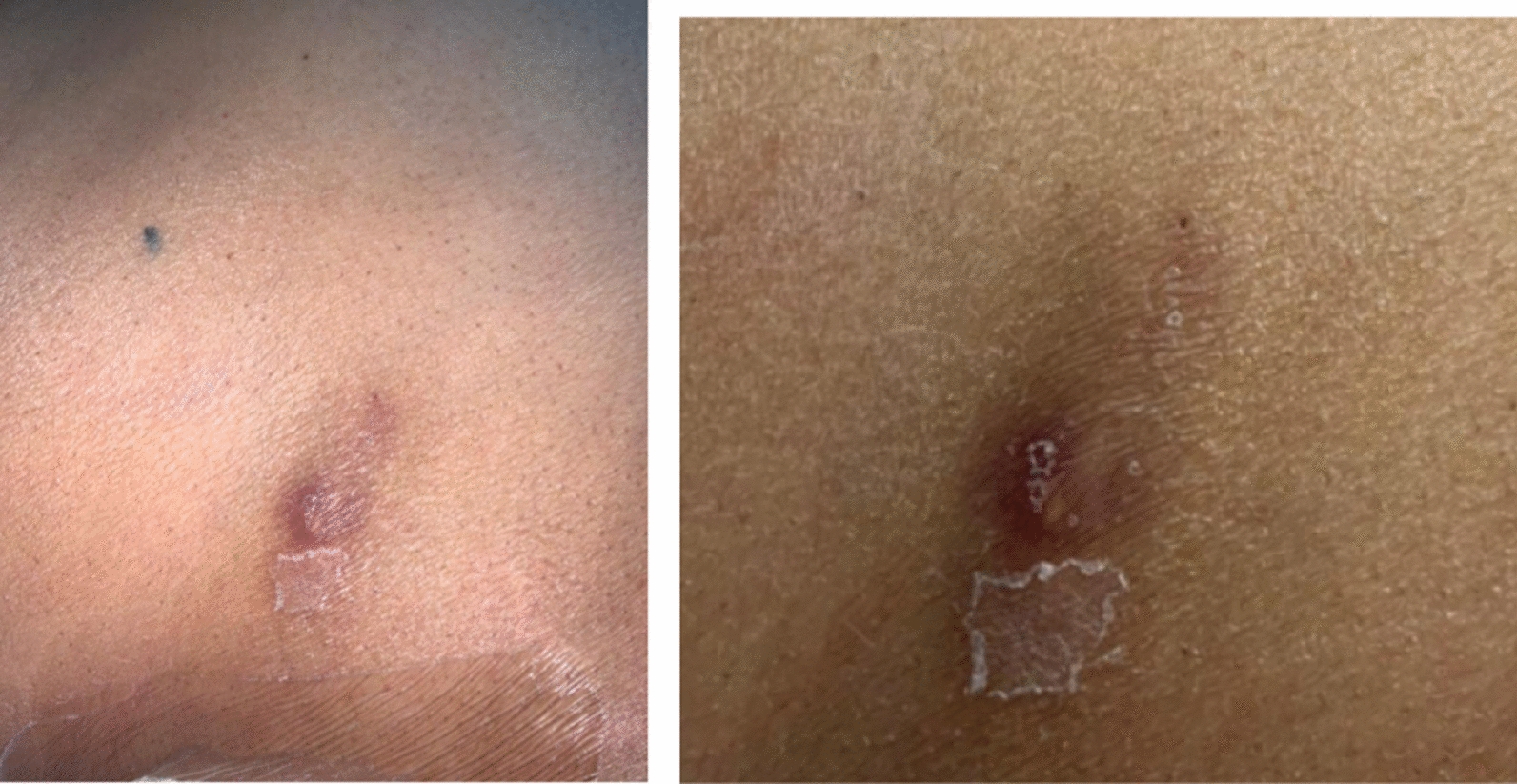
Nodular skin lesion overlying patient’s abdomen (left) and leg (right)

On arrival the patient’s vital signs were normal (Table 1), and her neurological examination was unremarkable. There were multiple nodules in the extensor area of the right leg and on the surface of her abdomen measuring 1 × 1 cm in size, they were firm, non-mobile with an erythematous base and overlying dark discoloration, they were mildly tender without any drainage (Fig. 1).

Blood results revealed elevated erythrocyte sedimentation rate (Table 1).

**Table 1:** Clinical and laboratory variables

Laboratory variable	Measurements	Normal value	Clinical variable	Measurements
WBC	9 × 10^9^/L	3.5–12.0 × 10^9^/L	Glasgow coma scale	15/15
Haemoglobin	125 g/L	120–160 g/L	Temperature	37 °C
Haemaocrit %	38%	37–47%	Blood pressure	110/64 mmHg
Platelets	144 × 10^9^/L	140–450 × 10^9^/L	Respiratory rate	20 Breaths/minute
International normalized ratio (INR)	0.93 s	0.8–1.3 s	Heart rate	88 Beats/minute
Erythrocyte sedimentation rate (ESR)	120 mm/h	0–29 mm/h	Oxygen saturation	99%
C-reactive protein	2.9 mg/L	< 10 mg/L		
Creatinine	55 µmol/L	53–115 µmol/L		

**Fig. 2:**
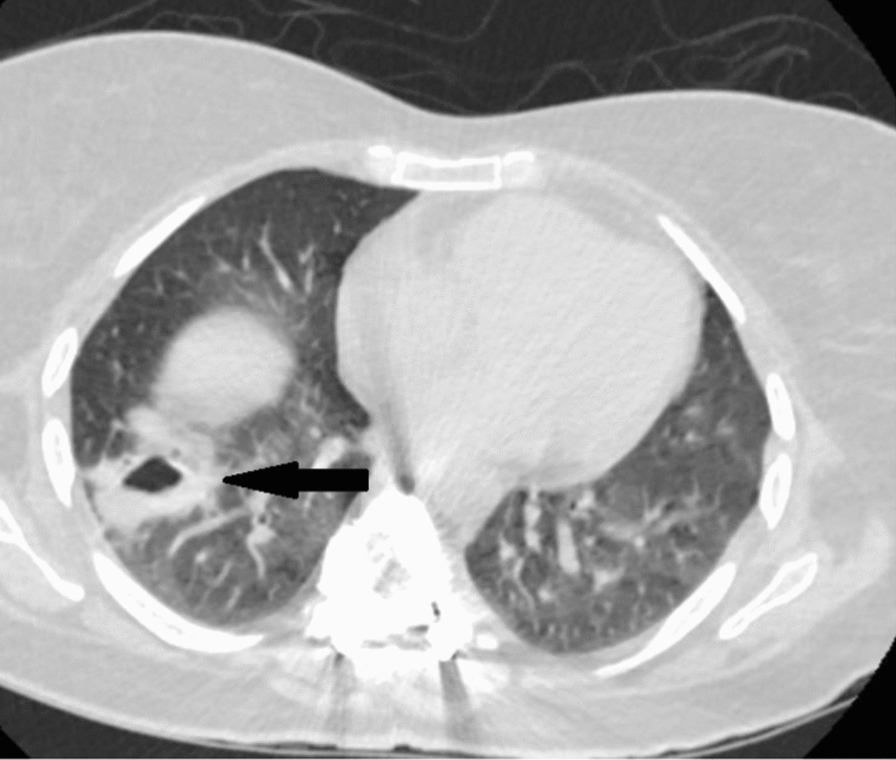
Axial CT chest with right lower lobe cavitary lesion containing air-fluid level (arrow)

Computed tomography (CT) of the chest showed right lower lobe consolidation with cavity containing air-fluid level, with bibasilar atelectatic bands (Fig. 2).

**Fig. 3:**
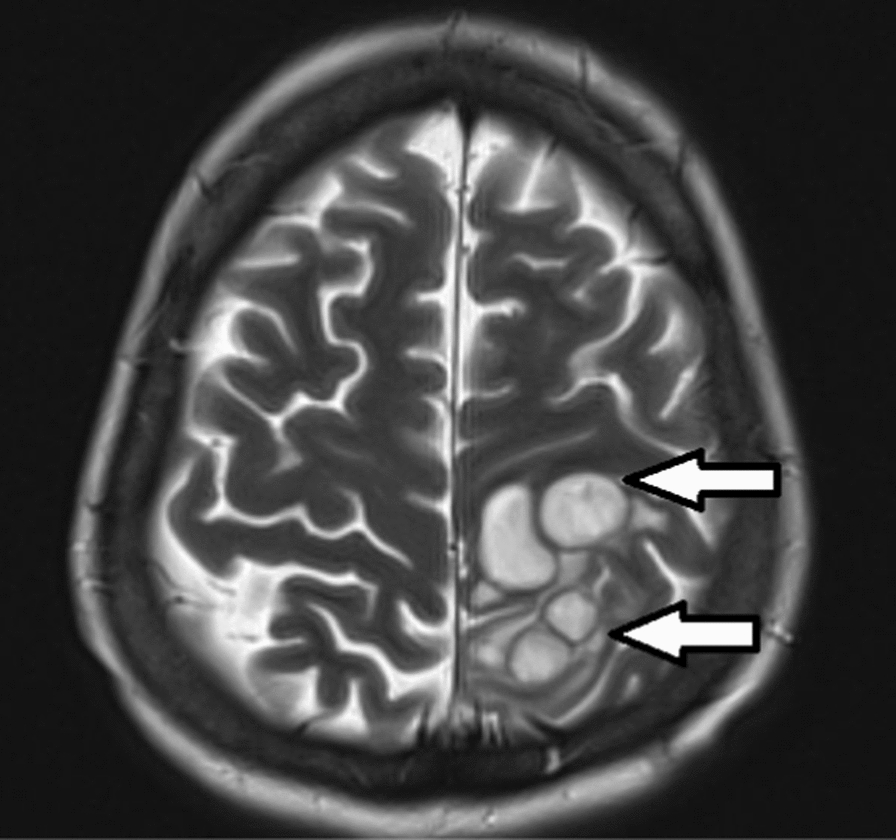
MRI T2 weighted image of brain showing cortical and subcortical cystic lesions with surrounding vasogenic edema in the left parietal lobe (arrows)

CT brain showed left parietal–temporal hypodensities. Cerebrospinal fluid (CSF) analysis showed no signs of inflammation and microbiological evaluation including *Mycobacterium tuberculosis* PCR did not reveal any pathogen. Magnetic resonance imaging (MRI) of the Brain showed multiple ring enhancing lesions in the left frontoparietal region, bilateral frontal regions, right parietal lobe and vermis, while T2 weighted image showed cortical and subcortical cystic lesions (Fig. 3).

**Fig. 4:**
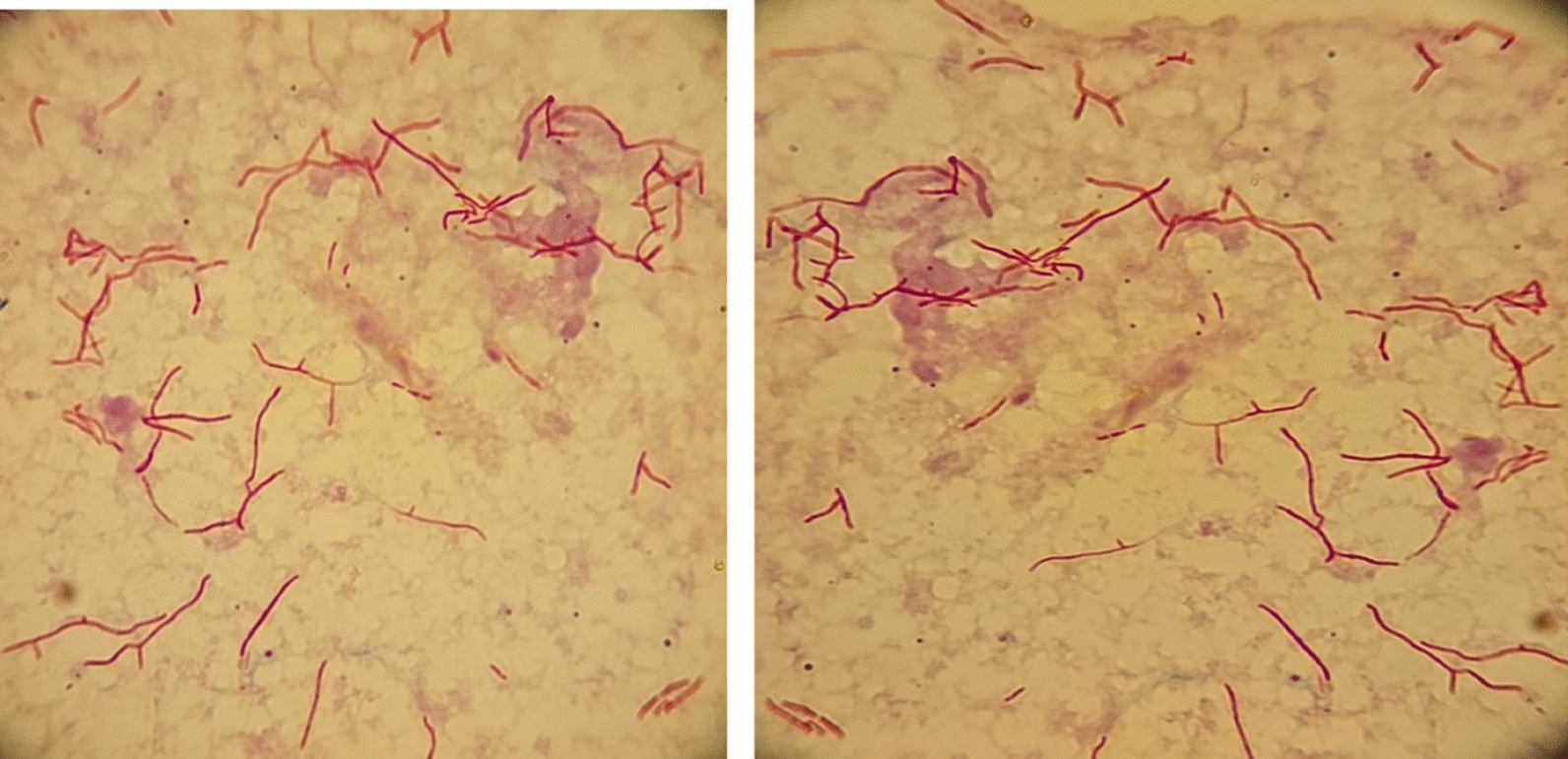
Brain tissue: modified Kinyoun stain with branching bacilli

A left parietal mini-craniotomy was performed and frank pus was drained with necrotic tissue. The histopathology showed no evidence for malignancy (metastasis) but histological features consistent with brain abscess and no granulomas. A modified Kinyoun stain showed branching bacilli (Fig. 4). The skin lesion punch biopsy modified Kinyoun stain showed similar result.

Tissue samples from the patient’s brain and skin lesions were cultured onto different media including sheep blood agar, chocolate agar, MacConkey agar and Sabouraud Dextrose Agar (Saudi Prepared Media Laboratory Company Ltd, Riyadh, Saudi Arabia). Initial growth was observed from both samples after 2 days of incubation on blood, chocolate and Sabouraud agars. There was only one colony type observed, the isolate was found to be catalase positive, the colony Gram showed beaded branching Gram-positive bacilli, acid fast stain was negative, and modified Kinyoun stain was positive. The organism was identified using Matrix-assisted laser desorption ionization-time of flight mass spectrometry (MALDI-TOF MS) (bioMerieux, Marcy-l ’Etoile, France) with 99.9% confidence value as *Nocardia otitidiscaviarum*. This was done through an extraction process using the Vitek MS *Mycobacterium/Nocardia* kit (bioMerieux, Marcy-l ’Etoile, France). Susceptibility testing was referred out and performed using the Sensititre™ RAPMYCOI panel (Thermo Fisher Scientific, Massachusetts, United States) and interpreted using Clinical and Laboratory Standards Institute interpretive breakpoints (CLSI M24: Clinical and Laboratory Standards Institute standard M24—Susceptibility Testing of *Mycobacteria*, *Nocardia* spp., and Other Aerobic Actinomycetes).

Upon presentation she was started on Imipenem-Cilastatin 500 mg IV every 6 h and trimethoprim–sulfamethoxazole (TMP–SMX) 15 mg/kg/day of TMP component IV divided every 8 h, on day 14 of hospital stay she developed a new skin lesion in the abdomen, Amikacin 30/kg/day IV was added. On day 22 of hospital stay, the susceptibility results returned from the outside referred lab showing TMP-SMX and Imipenem resistance (Table 2).

**Table 2:** Broth microdilution antibiotic susceptibility testing and interpretation based on CLSI M24

Antibiotic	MIC (μg/mL)	Interpretation
Trimethoprim–sulfamethoxazole	≥ 8/152	Resistant
Linezolid	4.0	Sensitive
Ciprofloxacin	≥ 4.0	Resistant
Imipenem	≥ 64.0	Resistant
Moxifloxacin	2.0	Intermediate
Cefepime	≥ 32.0	Resistant
Cefoxitin	128	Resistant
Amoxicillin–Clavulanic acid	≥ 64/32	Resistant
Amikacin	2.0	Sensitive
Ceftriaxone	16.0	Intermediate
Doxycycline	2.0	Intermediate
Minocycline	2.0	Intermediate
Tigecycline	0.5	Not applicable
Tobramycin	≥ 16.0	Resistant
Clarithromycin	≥ 16.0	Resistant

## Discussion and conclusion

In this report, we describe a rare fatal case of disseminated nocardiosis due to *N. Otitidiscaviarum* resistant to TMP–SMX in a lady with advanced cancer.

The most common clinical presentation of nocardial disease is pulmonary due to inhalation of mycelial fragments or via contact with the bacteria by a cut or abraded skin which may lead to extrapulmonary dissemination [1]. In our case, the patient’s risk factor was gardening.

*N. otitidiscaviarum*, formerly called *N. caviae*, was first reported in humans in the mid-1960s [2]. It was previously reported in 1924 after the organism was isolated from a guinea pigs middle ear [3]. Infections with *Nocardia* are being increasingly recognized, however, infections due to *N. otitidiscaviarum* are reported in only 0.3–2.9% of all *Nocardia*. infections and remains infrequently reported [4]. In one review only 10 cases of 347 patients infected with *Nocardia.* in the United States were identified as *N. otitidiscaviarum *[5]. In a Japanese report of more than 303 pathogenic *Nocardia* isolated from infected patients between 1992 and 2001, only 14 cases were due to *N. otitidiscaviarum* [6]. More recently, Chen Liu et al. described a fatal case of severe pneumonia due to *N. otitidiscaviarum* in an immunocompetent cotton farmer [7], while Ranjit Sah et al. reported successful treatment of a patient under steroid therapy with disseminated *N. otitidiscaviarum* [8]. Moreover, it was reported to cause disease in both immunocompetent and immunocompromised hosts in the forms of pulmonary, cutaneous, central nervous system and lymphocutaneous infections [4].

In nature, *N otitidiscaviarum* is found in soil, decomposing vegetation, and other organic matter, as well as in fresh and saltwater [9]. A survey of 504 soil samples in India revealed that *N*. *otitidiscaviarum* had a much lower prevalence compared to other *Nocardia* which may indicate the reason for its low incidence in clinical practice [10]. In addition, it was noted to be less pathogenic in humans when compared to other *Nocardia*.

Being described as an opportunistic pathogen, individuals with weakened immune system, such as patients suffering from diabetes mellitus, chronic obstructive pulmonary disease, mixed connective tissue disorder, ulcerative colitis, cirrhosis, human immunodeficiency virus (HIV) infection, malignancies, those receiving long-term or large doses of corticosteroid therapy, and stem cell or solid organ transplant recipients are at higher risk for infections due to *N. otitidiscaviarum * [11], similarly, our case had stage 4 invasive ductal carcinoma of the breast and was on chemotherapy for eight weeks with the last dose given three weeks before her presentation.

In a review of the database for *N. otitidiscaviarum*, 25 cases had been reported between 1997 and 2018 [7, 12–30]. More than half of those cases were reported in immunocompromised patients. Prolonged use of corticosteroids was a major risk factor in the majority [12, 13, 16, 20, 21, 23, 30, 31]. In other cases, organ transplant recipients [32], endocrine disorders [26], HIV and rheumatic heart disease were identified as risk factors [18, 20, 25]. On the other spectrum, eight cases were reported in immunocompetent patients. Four of those were engaged in gardening or farming and were exposed to dust inhalation similarly to our patient [7, 15, 19, 22]. Only one case reported no underlying immunocompromised state nor any occupational risk factors like farming [13].

It may be challenging to diagnose such patients who present with many possible differential diagnoses. Conventional evaluation of specimens including wound drainage, skin and brain lesions biopsies, CSF analysis and cultures along with imaging studies, all remain the principal diagnostic methods. In the present report, the species was determined using MALDI-TOF MS. Although the gold standard for *Nocardia* species identification is molecular biology with amplification and sequencing of one or two gene(s) among *rrs* (i.e. the gene coding for 16 S rRNA), *hsp65*, *secA1* and *sodA*, MALDI-TOF MS is increasingly being used for identifying *Nocardia* species. MALDI-TOF MS adequately identifies frequent species in 95–100% of cases, however for cases of a low identification score molecular biology-based identification remains important [33]. The isolate in the current study had a 99.9% confidence value, hence no molecular sequencing was performed.

Most *N. otitidiscaviarum* isolates are reported to be resistant to beta-lactams while usually being susceptible to Amikacin, Fluoroquinolones [12, 34], and trimethoprim–sulfamethoxazole, hence, Sulfonamides remain the standard agents for treatment [12]. Meanwhile, some studies reported *N. otitidiscaviarum* susceptibility to Linezolid *in-vitro*; however, data from *in-vivo* studies are still lacking and the risk of haematological toxicity with prolonged Linezolid therapy is high, hindering its clinical use [35, 36]. A study that assessed 552 clinical isolates of *Nocardia* from six major laboratories in the USA, found sulfonamide resistance to be only 2%, which is lower than previously shown [37, 38]. The authors hypothesized that these discrepancies may be associated with difficulty in the laboratory interpretation of in vitro MICs for TMP-SMX and SMX and the lack of quality controls for *Nocardia* for these agents [39]. The isolate in the current study showed MIC of ≥ 8/152 for TMP/SMX respectively, which indicates it to be a realistic phenomenon. However, Imipenem resistance has been more commonly described [34]. Typical *in-vitro* antimicrobial susceptibility patterns of various *Nocardia* species indicates that *N*. *otitidiscaviarum* is usually susceptible to TMP–SMX (Table 3).

**Table 3:** Typical *in-vitro* antimicrobial susceptibility patterns of various *Nocardia* species (Adapted from Manual of clinical microbiology [40])

Drug	*N. abscessus*	*N. brasiliensis*	*N. brevicatena* and *N. paucivorans*	*N. cyriacigeorgica*	*N. farcinica*	*N. nova* complex	*N. otitidiscaviarum*	*N. pseudobrasiliensis*	*N. transvalensis* complex
Amoxicillin–Clavulanic Acid	S	S	S	R	–	R	R	R	–
Amikacin	S	–	S	S	S	S	S	–	R
Ceftriaxone	S	–	S	S	R	S	R	–	S
Ciprofloxacin	R	R	S	R	S	–	S	S	S
Clarithromycin	R	R	R	R	R	S	–	S	R
Gentamicin	–	–	R	–	R	–	S	–	R
Imipenem	R	–	R	S	S	S	R	–	S
Linezolid	S	S	S	S	S	S	S	S	S
Minocycline	–	S	–	–	–	–	–	R	–
Sulfamethoxazole	–	S	–	–	–	–	S	S	–
Tobramycin	–	–	–	–	R	–	–	–	R

*S* susceptible, *R* Resistant

The optimal antimicrobial management for *N. otitidiscaviarum* is still not clearly defined, however, combined treatment is suggested for disseminated and severe disease. In our case, the isolated *N. otitidiscaviarum* was susceptible to Linezolid and Amikacin and was resistant to TMP-SMX, Ciprofloxacin, Imipenem, Cefepime, Cefoxitin, Amoxicillin-Clavulanic acid, Clarithromycin and Tobramycin. Seven cases were similarly reported with resistance to TMP-SMX [17, 19, 21, 24, 28, 30] with four cases reported in immunocompetent patients [17, 19, 24], and one infecting a farmer [19], details of these studies are described in Table 4

**Table 4:** Case studies of *N. otitidiscaviarum* resistant to TMP-SMX

References	Years	Age/gender	Immune Status	Drug Susceptibility	Treatment	Outcome
Matsu et al. [19]	2000	74M	Immunocompetent (Farmer)	Resistant to: TMP-SMX, Penicillin, Piperacillin, Imipenem, Ceftazidime. Susceptible to: Amikacin, Minocycline, Clarithromycin	TMP-SMX à Clarithromycin + Amikacin	Recovered
Yoshida et al. [30]	2004	69M	On Corticosteroid therapy	Resistant to: Ampicillin, Piperacillin, Cefazolin, Imipenem, minocycline, Vancomycin, TMP-SMX, Erythromycin. Susceptible to: Levofloxacin, Gentamicin and Levofloxacin	Imipenem + TMP-SMX à TMP-SMX + Gentamicin	Recovered
Mahgoub et al. [17]	2016	41F	Immunocompetent	Resistant to: Azithromycin, Ceftazidime, Penicillin, Rifampicin and TMP-SMX. Susceptible to: Amikacin, Ciprofloxacin, Meropenem and Streptomycin	TMP-SMX + Amikacin + Imipenem à Ceftriaxone + Amikacin + Ciprofloxacin	Recovered
Candel et al. [13]	2017	79M	On Corticosteroid therapy	Resistant to: TMP-SMX. Susceptible to Aminoglycosides, Beta-lactams and Carbapenem	Levofloxacin + Vancomycin + Tobramycin	Died
Princess et al. [21]	2018	51F	On Corticosteroid therapy	Resistant to: TMP-SMX, Amoxicillin, Clavulanate. Susceptible to: Amikacin, Ciprofloxacin, Linezolid, Imipenem and Ceftriaxone	Azithromycin + Doxycycline à TMP-SMX + Imipenem	Died
Saksena et al. [24]	2020	74M	Immunocompetent	Resistant to: Ampicillin, Amoxicillin-Clavulanate, Erythromycin, TMP-SMX and imipenem. SusceptibleTo: Amikacin, Linezolid, Ciprofloxacin, and Gentamicin	Amoxicillin-Clavulanate + Azithromycin à TMP-SMX	Died
Saksena et al. [24]	2020	74F	Immunocompetent	Resistant to: Ampicillin, Amoxicillin-Clavulanate, Erythromycin, TMP-SMX and imipenem. Susceptible to: Amikacin, Linezolid, Ciprofloxacin, and Gentamicin	Meropenem + Colistin à TMP-SMX added	Died

M, male; F, female; TMP-SMX, trimethoprim–sulfamethoxazole; GCS, Glasgow coma scale

Similarly, in our case combined drug therapy with Imipenem and TMP-SMX were used for a total of 22 days until the susceptibility result showed resistance to both agents and treatment with Linezolid, Ceftriaxone, moxifloxacin, doxycycline was used in addition to Amikacin that was added earlier due to the appearance of new skin lesions. The delay in obtaining susceptibility results from the outside lab clearly had a negative impact on patient outcome, such cases would advocate for wider availability of such testing and higher turnaround time.

Mortality due to disseminated Nocardiosi*s* is high, therefore early diagnosis and initiation of therapy are of vital importance in Nocardial infections. In our case, the patient was started on empirical treatment for Nocardiosis that was later adjusted according to the susceptibility results, however, the delay in administering proper antimicrobials and her pre-existing advanced malignancy may have attributed to her mortality.

In conclusion, *N. otitidiscaviarum* infection though rare may have considerable mortality; early diagnosis and susceptibility testing are crucial in avoiding similar devastating outcomes. In addition, surveillance for emerging TMP-SMX resistance should be closely monitored.

## Data Availability

All the data for this study will be made available upon reasonable request to the corresponding author.

## Abbreviations

TMP–SMX: Trimethoprim–sulfamethoxazole
WBC: White blood cells
ESR: Erythrocyte sedimentation rate
CRP: C-reactive protein
CT: Computed tomography
CSF: Cerebrospinal fluid
AFB: Acid-fast bacilli
M. tb: Mycobacterium tuberculosis
PCR: Polymerase chain reaction
MRI: Magnetic resonance imaging
GMS: Grocott methenamine silver
PAS: Periodic acid–Schiff
MALDI-TOF MS: Matrix-assisted laser desorption ionization-time of flight mass spectrometry
MIC: Minimum inhibitory concentration
CLSI M24: Clinical and Laboratory Standards Institute standard M24—Susceptibility Testing of Mycobacteria, *Nocardia* spp., and Other Aerobic Actinomycetes.
HIV: Human immunodeficiency virus
